# Ultra-Highly Sensitive Hydrogen Chloride Detection Based on Quartz-Enhanced Photothermal Spectroscopy

**DOI:** 10.3390/s21103563

**Published:** 2021-05-20

**Authors:** Yufei Ma, Ziting Lang, Ying He, Shunda Qiao, Yu Li

**Affiliations:** National Key Laboratory of Science and Technology on Tunable Laser, Harbin Institute of Technology, Harbin 150001, China; 20b921021@stu.hit.edu.cn (Z.L.); hearkenyi@hit.edu.cn (Y.H.); 18S021047@stu.hit.edu.cn (S.Q.); 19S021032@stu.hit.edu.cn (Y.L.)

**Keywords:** quartz tuning fork (QTF), quartz-enhanced photothermal spectroscopy (QEPTS), hydrogen chloride (HCl), trace gas detection

## Abstract

Combining the merits of non-contact measurement and high sensitivity, the quartz-enhanced photothermal spectroscopy (QEPTS) technique is suitable for measuring acid gases such as hydrogen chloride (HCl). In this invited paper, we report, for the first time, on an ultra-highly sensitive HCl sensor based on the QEPTS technique. A continuous wave, distributed feedback (CW-DFB) fiber-coupled diode laser with emission wavelength of 1.74 µm was used as the excitation source. A certified mixture of 500 ppm HCl:N_2_ was adapted as the analyte. Wavelength modulation spectroscopy was used to simplify the data processing. The wavelength modulation depth was optimized. The relationships between the second harmonic (2*f*) amplitude of HCl-QEPTS signal and the laser power as well as HCl concentration were investigated. An Allan variance analysis was performed to prove that this sensor had good stability and high sensitivity. The proposed HCl-QEPTS sensor can achieve a minimum detection limit (MDL) of ~17 parts per billion (ppb) with an integration time of 130 s. Further improvement of such an HCl-QEPTS sensor performance was proposed.

## 1. Introduction

Hydrogen chloride (HCl) is an essential gas used in chemical production which performs key roles in various important fields, for example, semiconductor manufacturing, biofuel combustion, and plasma etching [1,2]. The sources of atmospheric HCl are primarily incineration plants, accidental emission, and medical waste [3,4,5]. Considering that HCl is toxic, corrosive, and harmful to human health and the environment, there is significant demand for the detection of HCl gas concentration levels [6,7]. To continuously monitor and control concentration levels, sensors for HCl need to provide real-time data, fast response, and have a sensitive detection ability at low parts per million (ppm) and parts per billion (ppb) levels [5]. 

Various types of HCl sensors, including amperometric, optical, and solid electrochemical, have been investigated [1,2,3,4,5,6,7]. Laser absorption spectroscopy (LAS) with advantages of rapid response, as well as non-invasive, highly sensitive, and selective detection is widely applied for HCl sensing. Tunable diode laser absorption spectroscopy (TDLAS) has been adopted for measuring HCl and a precision of 2 ppm at 1 s averaging time has been obtained [8]. Generally, a multi-pass gas cell has been employed in TDLAS to improve the detection performance; however, this has resulted in the TDLAS sensor being bulky and costly. Quartz-enhanced photoacoustic spectroscopy (QEPAS) is another effective method for trace gas detection [9,10,11]. Due to the advantages of low cost (<USD 1), tiny volume (a few cubic millimeters), dipole structure, and high dynamic range and wavelength independence of the quartz tuning fork (QTF) [12,13,14,15,16,17], QEPAS has many merits such as low budget, compact size, and high sensitivity [18,19,20,21]. A minimum detection limit (MDL) of 550 ppb for HCl has been achieved by the QEPAS method [7]. However, owing to the fact that the QTF must be immersed in the target gas in QEPAS, the metal films on the surface of the QTF can be corroded during long-term exposure. The QTF resonance properties can be deteriorated by acid and corrosive gases such as HCl, which brings sensor failure. Therefore, achieving a non-contact measurement is especially essential for the detection of acid and corrosive gases.

Quartz-enhanced photothermal spectroscopy (QEPTS), another QTF-based sensitive trace gas detection technique, was invented, in 2018, by Ma et al. [22]. The laser first passes through the target gas, and then the gas absorbs partial energy of the laser. When the laser arrives at the QTF, the laser energy is absorbed by the QTF and converted into thermal energy [23,24]. Because of thermoelastic expansion, the thermal energy is translated into mechanical motion, which is enhanced by the resonant property of the QTF [25,26]. The mechanical motion creates an electrical signal due to the piezoelectric effect. By demodulating this electrical signal, the concentration of target gas can be retrieved. As compared with QEPAS, QEPTS has the same merits but also ensures the analyte does not contact the QTF, which means that QEPTS is suitable for acidic and corrosive gases detection. However, until now, HCl detection based on the QEPTS technique has not been reported.

In this invited paper, an ultra-highly sensitive HCl sensor based on the QEPTS technique was demonstrated for the first time. The proposed HCl-QEPTS sensor achieved an MDL of 17 ppb when the data acquisition time was 130 s. The reported HCl sensor is suitable for atmospheric monitoring and other applications that demand high sensitivity and long-term stability.

## 2. Experimental Setup

### 2.1. Absorption Line Selection

**Figure 1:**
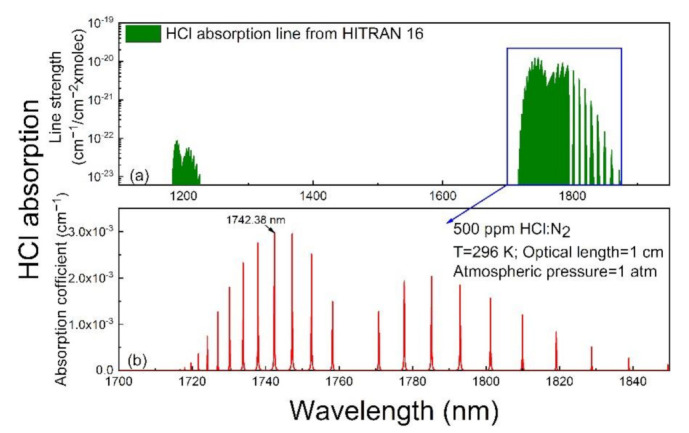
Simulation of HCl absorption based on the HITRAN 2016 database. (**a**) Absorption line strength; (**b**) absorption coefficient, at 296 K, standard atmospheric pressure, and an optical path length of 1 cm for 500 ppm HCl:N_2_.

As compared with mid-infrared lasers such as a quantum cascade laser (QCL), diode lasers have the advantages of compactness, low cost, and a fiber-coupled structure. Therefore, a diode laser with an output wavelength less than 3 µm was chosen as the light source. The absorption lines located below 3 µm were calculated according to the HITRAN 2016 database [27] and are shown in Figure 1a. It can be observed that absorption lines around 1.8 µm have stronger absorption line strength than those located at 1.2 µm. Therefore, as shown in Figure 1b, an intense absorption line located at 5739.27 cm^−1^ (1742.38 nm) was selected in this experiment.

### 2.2. Sensor Configuration

**Figure 2:**
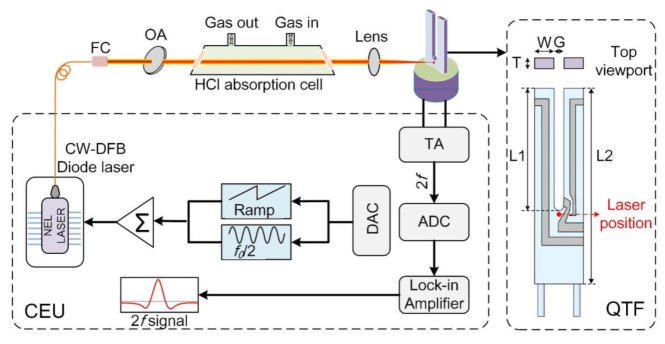
Schematic diagram of the experimental setup. FC, fiber collimator; OA, optical attenuator; TA, trans-impedance amplifier; ADC, analog-to-digital converter; DAC, digital-to-analog converter; CEU, control electronics unit. L1 = 3.9 mm, L2 = 5.4 mm, W = 0.62 mm, T = 0.36 mm, and G = 0.32 mm. The red dot indicates the optimum position for the laser beam on the QTF in QEPTS.

The experimental setup of the reported HCl sensor based on the QEPTS technique is shown in Figure 2. The laser excitation source was a continuous wave, distributed feedback (CW-DFB) fiber-coupled diode laser with a wavelength of 1.74 µm and optical power of 8 mW. A fiber collimator (FC) was used to collimate the laser beam which was sent into a HCl absorption cell with 20 cm length. The cell was equipped at both ends with a wedged CaF_2_ window to avoid optical interference. After passing through the cell, using a lens with 50 mm focal length, the laser beam was focused on the root of the QTF, which is shown with a red dot in Figure 2, to generate the strongest photothermal signal [28]. The dimensions of the QTF’s prongs are shown in the enlarged view of Figure 2. The laser power was changed by employing an optical attenuator (OA) to investigate the power response of the HCl-QEPTS sensor. Because the integration time of the QTF can be increased by using a QTF with low resonance frequency (*f*_0_) [29], therefore, a QTF with an *f*_0_ of 30.72 kHz (in vacuum) was employed in the experiment to improve the QEPTS sensor performance. The geometry of the used QTF is depicted in Figure 2. Wavelength modulation spectroscopy (WMS) and second harmonic (2*f*) detection were utilized to detect the electrical signals. A ramp with low frequency and a sinusoidal wave with a high frequency of *f*_0_/2 were added together to scan and modulate the wavelength of the used CW-DFB diode laser. The piezoelectric signal generated by the QTF was obtained by a custom control electronics unit (CEU). A CEU contains six parts, including a trans-impedance amplifier (TA), analog-to-digital converter (ADC), lock-in amplifier, digital-to-analog converter (DAC), adder, and diode laser. By using CEU, the demodulated 2*f* component of QEPTS was acquired. The integration time of lock-in amplifier for the HCl-QEPTS sensor was 1 s. A certified mixture of 500 ppm HCl:N_2_ was adapted as the analyte. The measurements were carried out at atmospheric pressure and room temperature.

## 3. Experimental Results and Discussions

**Figure 3:**
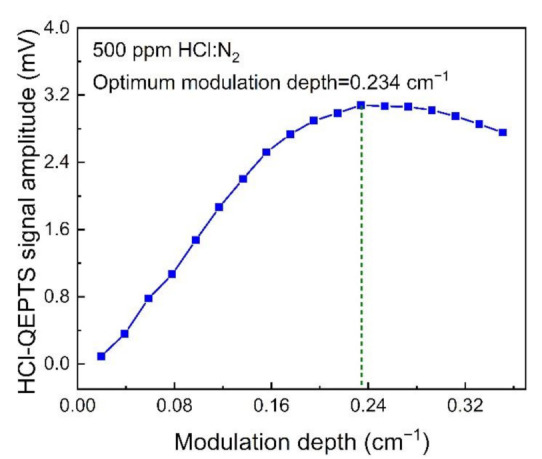
HCl-QEPTS 2*f* signal amplitude as a function of the modulation depth. The blue solid line is a guide for visually connecting the experimental data points. The green dotted line is used to mark the optimum modulation depth.

Firstly, the dependence of the HCl-QEPTS signal amplitude on wavelength modulation depth was investigated and the obtained results are shown in Figure 3. It can be observed that the HCl-QEPTS signal amplitude first increased, and then decreased with an increase in modulation depth. The maximum signal value was achieved when the modulation depth was 0.234 cm^−1^. Therefore, the optimized modulation depth was found to be 0.234 cm^−1^.

**Figure 4:**
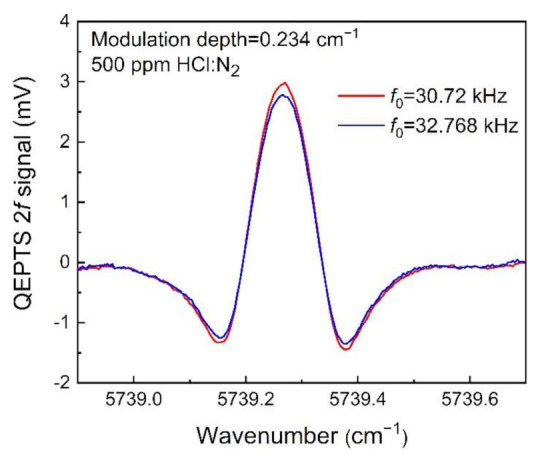
The QEPTS 2*f* signals for the two QTFs. The resonance frequencies of the two QTFs are 30.72 and 32.768 kHz (in vacuum), respectively.

To investigate the relationship between HCl-QEPTS 2*f* signal amplitude and the QTF, two different QTFs with *f*_0_s of 30.72 and 32.768 kHz (in vacuum) were tested. The properties of the QTF were investigated by using the CEU, and they are listed in Table 1. The equivalent resistance (*R*), quality factor (*Q*), and *f*_0_ for the first QTF with an *f*_0_ of 30.72 kHz (in vacuum) were measured as 134 kΩ, 12,100, and 30.70 kHz, respectively. For the second QTF with an *f*_0_ of 32.768 kHz (in vacuum), the measured results of *R*, *Q*, and *f*_0_ were 84.6 kΩ, 14,405, and 32.763 kHz, respectively. A low *f*_0_ is better to accelerate the relaxation rate of target gas. The *R* refers to the charge loss in the equivalent resonator circuit. A low *R* can enhance charge generation capability. The *Q* is affected by all the energy dissipation mechanisms around the vibrating QTF prong. A high *Q* is beneficial to the signal amplitude and detection sensitivity [30]. The QEPTS 2*f* signals for the two QTFs are shown in Figure 4. From this figure, it can be observed that the QEPTS 2*f* signal amplitude for the QTF with an *f*_0_ of 30.72 kHz is higher than the QTF with an *f*_0_ of 32.768 kHz, which confirmed the advantages of using the QTF with a low *f*_0_ of 30.72 kHz.

**Figure 5:**
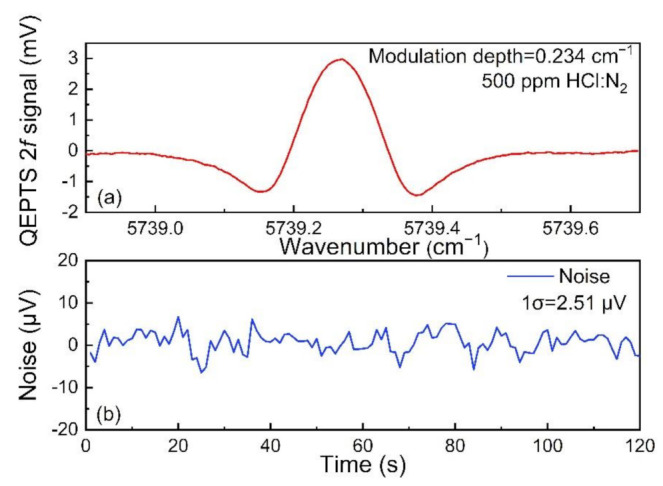
The 2*f* signal and noise for the HCl-QEPTS sensor. (**a**) QEPTS 2*f* signal with laser power of 8 mW; (**b**) noise level determination when pure N_2_ was adopted.

The HCl-QEPTS 2*f* signal was measured, as shown in Figure 5a, when the laser modulation depth was 0.234 cm^−1^ and incident laser power was 8 mW. The maximum signal amplitude was 2.99 mV. The background noise level was determined by continually monitoring the amplitude while the laser wavelength was located at the HCl absorption line for 120 s with pure nitrogen (N_2_) filled into the HCl absorption cell. The results are depicted in Figure 5b, and the calculated 1σ noise was 2.51 µV. On the basis of the above results, a signal-to-noise ratio (SNR) value of ~1191 was calculated for this HCl-QEPTS sensor. According to the formula of minimum detection limit, (MDL) = analyte concentration/SNR, an MDL of ~419.8 ppb was obtained.

**Figure 6:**
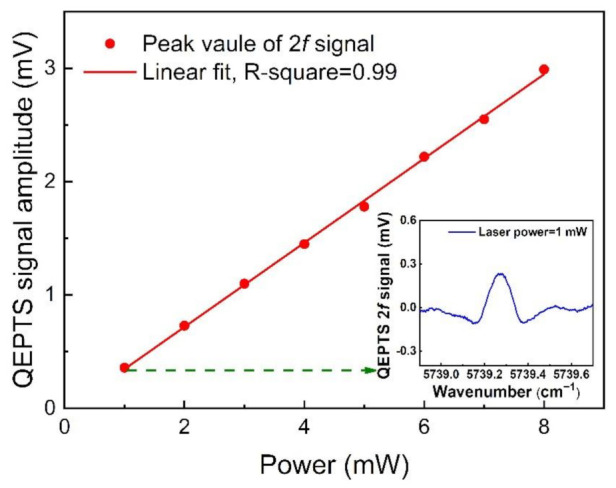
HCl-QEPTS sensor 2*f* signal amplitude as a function of laser power at the optimized modulation depth. The insert shows QEPTS 2*f* signal with laser power of 1 mW.

To investigate the relationship between laser power and the HCl-QEPTS signal amplitude, an adjustable OA was placed between the FC and HCl absorption cell to change the power from 1 to 8 mW. The measured HCl-QEPTS signal amplitude as a function of the laser power was plotted, as shown in Figure 6. The linear fitting based on signal amplitude and laser power was established. The R-square for the fitting was equal to ~0.99, which indicated that laser power and signal amplitude had a good linear relationship. A 2*f* signal waveform was inserted in Figure 6 when the laser power was 1 mW. The HCl-QEPTS sensor signal level can be increased when a laser with high optical power is used.

**Figure 7:**
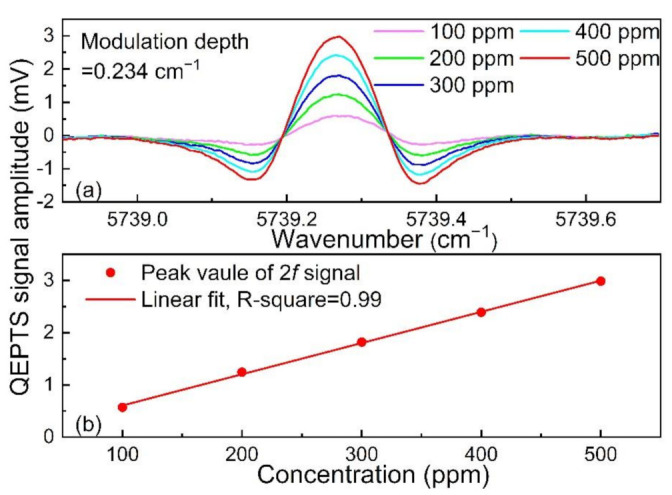
Variation of QEPTS 2*f* signal with HCl concentrations. (**a**) QEPTS 2*f* signals at different HCl concentrations with modulation depth of 0.234 cm^−1^; (**b**) peak value of QEPTS 2*f* signal at different HCl concentrations and corresponding linear fitting.

To verify the linear response of QEPTS signal and HCl concentration, the QEPTS 2*f* signal for different HCl concentrations was investigated, as shown in Figure 7a. The gas flow rate of a mixture of 500 ppm HCl:N_2_ and pure dry N_2_ were controlled by two mass flow controllers to obtain different HCl concentrations. The linear fit of the QEPTS signal amplitude and HCl concentration are shown in Figure 7b, and the value of R-square was equal to ~0.99. These results imply that the HCl-QEPTS sensor has an excellent linear response, which is convenient for practical applications in HCl detection.

**Figure 8:**
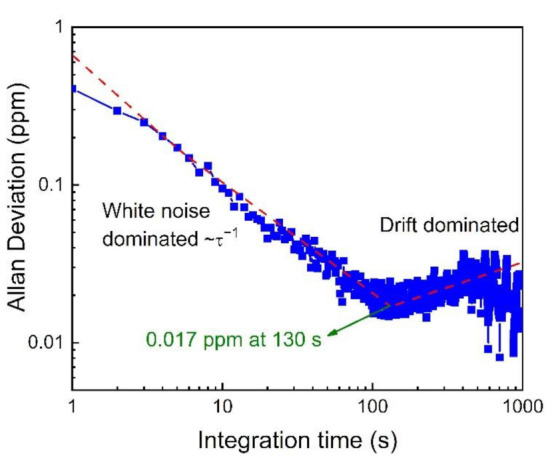
Allan deviation analysis for the HCl-QEPTS sensor. The minimum in the Allan deviation is determined by using the fitting of ~τ^−1^ function for the experimental data.

To investigate the long-term stability of this HCl-QEPTS sensor, an Allan variance analysis was performed by measuring and averaging the data using pure N_2_. The results are displayed in Figure 8. When white noise dominates the measurements, the Allan plot follows the ~τ^−1^ slope. A fitting using ~τ^−1^ function for the partial experimental data was done in the Allan deviation. The minimum in the Allan deviation corresponds to the detection limit at the optimum integration time. As a result of system instability, sharp spikes appeared after 400 s. It was observed that when the optimum integration time was 130 s, the MDL of ~17 ppb could be achieved. In addition, good stability of the HCl-QEPTS sensor was proven, according to the long optimum integration time of 130 s. 

## 4. Conclusions

In this paper, we demonstrate, for the first time, an ultra-highly sensitive QEPTS sensor for HCl detection. A CW-DFB fiber-coupled diode laser with an emission wavelength of 1.74 µm was used as the excitation source. A QTF with an *f*_0_ of 30.70 kHz was employed as a photothermal detector. A certified mixture of 500 ppm HCl:N_2_ was adapted as the analyte. The optimized wavelength modulation depth was 0.234 cm^−1^. By calculating the measured 2*f* signal and noise, an MDL of ~419.8 ppb was obtained when the integration time was 1 s. It was verified that this HCL-QEPTS sensor had a good linear response with the laser power and HCl concentration. An Allan variance analysis was performed to prove this sensor had excellent stability and high sensitivity. The proposed HCl-QEPTS sensor can achieve an MDL of ~17 ppb with an integration time of 130 s. As compared with traditional TDLAS and QEPAS methods, the proposed HCl-QEPTS sensor is low cost and ultra-highly sensitive, and the QTF is not corroded by HCl, therefore, improving system stability. Furthermore, increasing the absorption path and optical power are effective methods to enhance the photothermal signal of such a sensor.

## Figures and Tables

**Table 1:** The measured properties of the two QTFs in atmospheric pressure.

QTF	*f*_0_ (kHz)	*R* (kΩ)	*Q*
1	30.70	134	12,100
2	32.763	84.6	14,405

## Data Availability

The data presented in this study are available on request from the corresponding author.
